# Fluctuating vs. Continuous Exposure to H_2_O_2_: The Effects on Mitochondrial Membrane Potential, Intracellular Calcium, and NF-κB in Astroglia

**DOI:** 10.1371/journal.pone.0076383

**Published:** 2013-10-04

**Authors:** Aleksandar Bajić, Mihajlo Spasić, Pavle R. Andjus, Danijela Savić, Ana Parabucki, Aleksandra Nikolić-Kokić, Ivan Spasojević

**Affiliations:** 1 Center for Laser Microscopy, Faculty of Biology, University of Belgrade, Belgrade, Serbia; 2 Department of Physiology, Institute for Biological Research ‘Siniša Stanković’, University of Belgrade, Belgrade, Serbia; 3 Department of Neurobiology, Institute for Biological Research ‘Siniša Stanković’, University of Belgrade, Belgrade, Serbia; 4 Life Sciences Department, Institute for Multidisciplinary Research, University of Belgrade, Belgrade, Serbia; University of Tasmania, Australia

## Abstract

The effects of H_2_O_2_ are widely studied in cell cultures and other *in vitro* systems. However, such investigations are performed with the assumption that H_2_O_2_ concentration is constant, which may not properly reflect *in vivo* settings, particularly in redox-turbulent microenvironments such as mitochondria. Here we introduced and tested a novel concept of fluctuating oxidative stress. We treated C6 astroglial cells and primary astrocytes with H_2_O_2_, using three regimes of exposure – continuous, as well as fluctuating at low or high rate, and evaluated mitochondrial membrane potential and other parameters of mitochondrial activity – respiration, reducing capacity, and superoxide production, as well as intracellular ATP, intracellular calcium, and NF-κB activation. When compared to continuous exposure, fluctuating H_2_O_2_ induced a pronounced hyperpolarization in mitochondria, whereas the activity of electron transport chain appears not to be significantly affected. H_2_O_2_ provoked a decrease of ATP level and an increase of intracellular calcium concentration, independently of the regime of treatment. However, fluctuating H_2_O_2_ induced a specific pattern of large-amplitude fluctuations of calcium concentration. An impact on NF-κB activation was observed for high rate fluctuations, whereas continuous and low rate fluctuating oxidative stress did not provoke significant effects. Presented results outline the (patho)physiological relevance of redox fluctuations.

## Introduction

Under physiological settings, the intracellular concentration of H_2_O_2_ is kept low and tightly regulated [1]–[3]. However, in various non-physiological conditions, the generation of H_2_O_2_ by mitochondria, specific enzymes or non-enzymatic reactions might be deregulated, resulting in excessive oxidation, altered signaling, and damage of biomolecules. In relation to this, the effects of H_2_O_2_ on cellular processes are the subject of a large number of studies, many of which are performed on cell cultures. A spectrum of experimental settings has been intercrossed, differing in H_2_O_2_ concentrations, cell types, and measured parameters. Surprisingly however, one potentially important factor has remained overlooked and unexplored until now. Namely, the uncontrolled or even physiologically-stimulated production of H_2_O_2_ may lead to unstable or fluctuating H_2_O_2_ levels [1], [4], [5]. The level of H_2_O_2_ in the cell depends on the activity of different intracellular sources, the efficiency of antioxidative defense (catalase, glutathione peroxidase/reductase), as well as the H_2_O_2_ concentration in the extracellular compartment, since H_2_O_2_ is sufficiently stable to pass the cellular membrane [2]. Such a complex system, composed of various interactions, coupled biochemical reactions, and feedback loops, clearly may result in fluctuating H_2_O_2_ levels. It is worth mentioning that H_2_O_2_ concentration fluctuates in tissues with the circadian rhythm, according to the fluctuating patterns of catalase activity [6]. Additionally, the production of H_2_O_2_ may fluctuate at one source. For example, H_2_O_2_ is produced in mitochondria by manganese superoxide dismutase (MnSOD) from superoxide, and its generation depends on a set of variable parameters [7]. It has been documented that well-defined clusters of mitochondria show discrete bursts of superoxide (flashes) with specific frequency and amplitude. The number of active clusters is increased upon metabolic stimulation [8].

Some systems, such as CNS, might be particularly prone to H_2_O_2_ fluctuations. CNS shows very high oxygen turnover combined with a generally weak antioxidative system [9]. Nervous tissue spontaneously generates and releases H_2_O_2_ into the extracellular/cerebrospinal fluid, which is poor in antioxidants [10]. The concentration of H_2_O_2_ might reach up to 1 mM in the cerebrospinal fluid under physiological conditions, while in neurodegeneration the production is promoted via several different pathways [11]. These conditions can be mimicked *in vitro* by exogenously administered H_2_O_2_ [12]. It is of particular interest to elucidate the effects of H_2_O_2_ on astroglial cells in relation to their function as the antioxidative 'barrier' in CNS. Of all mammalian cells, neurons appear to show the most perilous O_2_ consumption/antioxidant level ratio, and it has been proposed that they are protected from H_2_O_2_ by astroglia [13]–[15]. Astroglial cells are comparatively resistant to oxidative stress, possessing specific protective mechanisms which enable them not only to survive pro-oxidative conditions and/or mitochondrial dysfunction, but also to metabolically support neurons in the time of crisis [16], [17]. Studies show that continuous exposure of astroglial cells to H_2_O_2_ alters NF-κB transcription [2], depletes intracellular ATP [18], [19], provokes an increase in [Ca^2+^]_i_ [20], and affects mitochondrial functions [21]–[23]. Pertinent to the latter, Buckman *et al.* have shown using three different MitoTracker™ dyes that the treatment of neurons and astrocytes with H_2_O_2_ results in concentration-dependent increase of mitochondrial membrane potential (MMP) [21]. We came up to a similar conclusion in our experiments, where we exposed astrocytes to H_2_O_2_ (3 mM) for 10 minutes, and used MMP-sensitive dye MitoTracker™ Orange (MTO) [22]. Finally, it has been shown that the hyperpolarization of mitochondria, which takes place in neurons exposed to supraphysiological H_2_O_2_ concentrations, is caused by the accumulation of H^+^ ions in the intermembrane space, and that it can be prevented by the inhibition of complex I [23].

The aim of our study was to explore a novel concept of fluctuating oxidative stress and to overcome the gap in the fundamental knowledge on the effects of H_2_O_2_. In order to compare the effects of three regimes of exposure to increased H_2_O_2_ concentration – continuous, fluctuating at low rate, and fluctuating at high rate, on C6 astroglial cells and primary astrocytes, we performed laser scanning confocal microscopy, intracellular ATP and calcium measurements, electron paramagnetic resonance (EPR) oximetry, MTT assay, mitochondrial superoxide production measurements, and NF-κB/p65 immunofluorescence experiments.

## Materials and Methods

### Chemicals

Chemicals of the highest grade available were obtained from commercial providers: H_2_O_2_ (Renal, Budapest, Hungary); culture medium RPMI-1640, ATP bioluminescence assay kit (Sigma-Aldrich, St. Louis, MO, USA); TrypLE™ Express, MTO, Hoechst 33258, DAPI, Alexa Fluor 555 donkey-anti-rabbit, MitoSOX™ Red, Fluo-3, streptomycin (Life Technologies - Invitrogen, Carlsbad, CA, USA); Dulbecco's modified Eagles's medium and heat-inactivated fetal calf serum (FCS) (PAA Laboratories GmbH, Cölbe, Germany); rabbit anti-NF-κB/p65 (sc-109; Santa Cruz, Dallas, TX, USA); Mowiol (Calbiochem - Millipore, Billerica, MA, USA); BCA kit (Pierce, Rockford, IL, USA); India ink (Talens, Apeldoorn, Netherlands; kindly provided by Tattoo Magic Studio, Belgrade); all other chemicals (Sigma-Aldrich).

### Cell cultures

Rat C6 astroglioma cells were cultured in 25 cm^2^ flasks at 37°C in a humidified atmosphere with 5% CO_2_ and passaged once a week. The culture medium was RPMI-1640 supplemented with NaHCO_3_ (2 g/L), glucose (10 mM), FCS (5%), and gentamicin (50 µg/mL). For the experiments, the cells were detached with TrypLE™ Express and seeded into 35-mm dishes at a density of 2×10^5^. The cells exhibited differentiated phenotype and formed a semiconfluent layer prior to the start of experiment.

Primary astrocytes were prepared from 3-days old Wistar pups in accordance with Institutional Animal Care and Use Committee Guideline. The study was approved by the Ethical Committee of Faculty of Biology, University of Belgrade. Animals were bred in-house under flow sterile conditions with free access to food and water. All efforts were made in order to minimize animal suffering and to reduce the number of animals used. Pups were anesthetized in a CO_2_ chamber for anesthesia, placed on ice, and sacrificed by decapitation. Cerebral cortices were dissected and meninges were peeled off in ice-cold phosphate-buffered saline (PBS). Tissue was subsequently mechanically dispersed in the culture medium, filtered through a 40-µm nylon mesh, and centrifuged at 400 g for 5 min. Culture medium was Dulbecco's modified Eagles's medium with the addition of FCS (10%), glucose (25 mmol/L), glutamine (2 mmol/L), sodium pyruvate (1 mmol/L), penicillin (100 IU/mL), and streptomycin (100 µg/mL). Resuspended mixed glial cells were seeded subsequently in 75 cm^2^ tissue culture flasks and grown at 37°C in a humified incubator with 5% CO_2_. Culture medium was replaced every third day. After 7–10 days in culture, confluence was reached and primary microglial cells and oligodendrocytes were removed by moderate shaking. Adherent primary astrocytes were washed with PBS, trypsinized (0.25% trypsin and 0.02% EDTA) and resuspended in the culture medium. Astrocytes were seeded into 35-mm dishes at a density of 10^5^ cells per dish and grown 2–3 days until confluence.

### Experimental design

Two to three days after seeding, cultures were washed with ECS (extracellular solution: NaCl (135 mM), KCl (5 mM), CaCl_2_ (2 mM), MgCl_2_ (1 mM), glucose (10 mM), HEPES (10 mM), bovine serum albumin (BSA) (0.1%); pH and osmolarity adjusted to pH 7.4 and 290±5 mOsm). The final volume of ECS was 1 mL per dish. In the next step, cultures were placed in gravity perfusion system (ALA VC3-8PG, HEKA Instruments Inc, Bellmore, NY, USA), which was adjusted to provide 3 mL/min flow. The samples were perfused with ECS containing H_2_O_2_ (3 mM final concentration) continuously for 10 min, or using two different fluctuating exposure sequences: Pulse 2 (2 min ECS + H_2_O_2_/2 min ECS/2 min ECS + H_2_O_2_/2 min ECS/2 min ECS + H_2_O_2_) or Pulse 1 (5×(1 min ECS + H_2_O_2_/1 min ECS)). Controls were perfused with ECS for 10 min. The treatment was conducted at room temperature. Samples were washed 3 times with ECS (without BSA) immediately after the treatment.

### Confocal microscopy

Fluorescence signals were recorded and analyzed using an LSM 510 confocal system (Carl Zeiss, Jena, Germany). Achroplan 40× objective with numerical aperture of 0.8 was used in order to obtain acquisitions of MMP from non-fixed cells. MTO was stimulated by 543 nm light, and the emission was collected with a long pass filter above 560 nm. Pinhole was adjusted to provide collection of light from 3.5 µm thick sections. Fluo-3 and MitoSOX fluorescence signals were activated by argon-ion 488-nm laser line and measured between 505–530 nm or above 585 nm, respectively.

MTO was diluted in ECS (BSA free) to the final concentration of 50 nM, and cultures were labeled at 37°C for 10 min. The cells were rinsed once again with ECS and left for 10 min in the dark to accommodate at room temperature before imaging. MTO was selected for this study because its fluorescence intensity in stained astrocytes shows a very consistent dependence on MMP and H_2_O_2_ concentration that was applied in the pre-treatment [21], [24], [25]. In addition, at the concentration applied here, MTO does not affect respiration rates [21]. At least five different fields were imaged from each dish. The recordings were conducted within 15 min. Mean per-pixel fluorescence intensities were evaluated from whole images after setting the threshold level to exclude ‘out-of-cell’ pixels. Fluorescence intensities of all images obtained from a dish were averaged, compared to mean control values that were obtained on the same experimental day, and presented as arbitrary units. In addition, the average per-pixel fluorescence intensity was determined for each cell in samples exposed to Pulse 2 and Pulse 1 regimes, using Image J (NIH, USA). This was done in order to establish the percentage distribution of cells showing fluorescence intensities within specific range (10 AU steps were used, *e.g.* 0–10 AU, 10–20 AU, etc). The whole range of fluorescence intensities (0–255 AU) was covered.

Superoxide production was evaluated using MitoSOX, while [Ca^2+^]_i_ was tracked with Fluo-3. The probes were loaded simultaneously to C6 cells. In brief, cells were incubated with Fluo-3 (5 µM) in ECS for 50 min, and then MitoSOX (5 µM) was added and the cells were incubated for additional 10 min (a total of 60 min for Fluo-3 staining). Excessive stains were removed by ECS replacement, and cells were left in the dark for 10 min. Subsequently, the perfusion was established throughout the dish and cells were imaged (every 3 s) with confocal imaging system for 5 min before the initiation of the treatment (in order to establish baseline fluorescence intensity), during 10 min treatment period, and 2 min after the end of the treatment. Results are presented as mean values from 20 regions of interest (cell bodies), normalized to the baseline per-pixel intensity for each cell.

### Total thiols quantification

The content of thiol groups in C6 cells was determined according to Ellman. At the end of treatment, cell cultures were snap frozen in liquid nitrogen and stored at −80°C. Frozen cells were detached with a Teflon cell scraper and dissolved in 1 mL of TRIS-HCl buffer containing 0.25 M sucrose and 1 mM EDTA (pH 7.4), and then sonicated and centrifuged (90 min/105000 g/4°C). The supernatant was used to determine the content of thiol groups (using a UV spectrophotometer, Shimadzu Scientific Instruments, Shimadzu Corporation, Kyoto, Japan) with Ellman's reagent [26]. The amount of thiols was expressed as µmol/g protein. Protein content was determined by the method of Bradford.

### MTT assay

MTT assay measures the reducing capacity of living cells. MTT reduction leads to precipitation of colored formazan. The experimental design was modified for experiments using 96-well plates. C6 cells were seeded in first 48 wells, at the density of 8000 cells/well and incubated for 48 h. The solutions were changed rapidly using 12-channel micropipettes (one row – one exposure sequence) in all wells every minute during 10 min period. Immediately following the treatment, MTT was added to cells at a final concentration of 5 mg/mL. After incubation at 37°C for 40 min, the resultant formazan crystals were dissolved in dimethyl sulfoxide (150 µL/well), and the absorbance was determined using microplate reader (LKB 5060-006, LKB Vertriebs GmbH, Vienna, Austria) at 492 nm. The results were normalized to mean control value obtained on the same experimental day.

### ATP assay

The extraction of ATP from cells was carried out using a one-step method with boiling water, as described previously [27]. In brief, ECS was aspirated promptly after each treatment, and cells were suspended in 400 µL of boiling deionized water by repeated pipetting. Cell suspensions were transferred into microtubes and boiled for 10 minutes. After cooling down on ice, suspensions were centrifuged at 12000 g/5 min/4°C, and supernatants were frozen and stored at −80°C. ATP concentration was measured using an ATP bioluminescence assay kit. Luciferin–luciferase agent (100 µL) was added to samples (100 µL), and ATP-dependent luminescence was measured with a luminometer (CHAMELEON™V, Hidex, Turku, Finland). The standard curve was obtained by serial dilutions of 10 µM ATP solution. ATP concentrations were expressed as nmol/mg of proteins, the amount of which was determined using BCA kit.

### EPR oximetry

Immediately after the treatment, cells were washed with culture medium, detached from the dish with TrypLE™ Express, centrifuged at 400 g/5 min and suspended in 120 µL of culture medium, and pO_2_-sensitive paramagnetic probe – India ink (5 µL) was added. Samples were placed in EPR quartz flat cell and sealed from air with Teflon plugs. Special care was taken in order to eliminate any air bubbles from the sample. EPR spectra were recorded at room temperature every 5 min for 1 h (the first spectrum was recorded 15 min after the treatment), using a Varian E104-A EPR spectrometer (Palo Alto, CA, USA) operating at X-band (9.54 GHz) with the following settings: scan range, 40 G; modulation amplitude, 1 G; modulation frequency, 100 kHz; microwave power, 20 mW; time constant, 128 ms; scanning time, 4 min. Oxygen tensions were calculated from the line widths of India ink spectra with reference to the calibration of India ink in water against known pO_2_. India ink was selected for this study because the line width of its spectrum is particularly sensitive to pO_2_ in the region of biological interest (1–40 mmHg), and because it is non-toxic, very stabile, resistant to oxidation and reduction, and insensitive to pH and spin-spin broadening [28], [29].

### NF-κB/p65 immunofluorescence and quantification

The effects of H_2_O_2_ on NF-κB were determined by measuring nuclear fluorescence intensity of NF-κB/p65 subunit. For immunofluorescence imaging, C6 cells were seeded into glass bottom 35-mm dishes. Following the treatment, cells were incubated for 2 h at 37°C and fixed in PFA (4%) at 4°C for 20 min. Cells were then washed with PBS and permeabilized in Triton X-100 (0.25%) for 10 min, which was followed by the blocking of non-specific staining in BSA (3%) for 30 min. Then, cells were stained with rabbit anti-NF-κB/p65 (1∶100) over night and visualized with fluorophore-labeled secondary antibody (Alexa Fluor 555 donkey-anti-rabbit, 1∶100). Cell nuclei were counterstained with Hoechst 33258 and cover-slipped with Mowiol. Images were acquired using Zeiss Axiovert microscope. Fluorescence measurements were performed using Image J, as described previously [30]. Total fluorescence intensity of nuclear NF-κB/p65 was measured in 6 fields (0.38 mm^2^; each field contained around 30 cells) per each experimental group.

### Statistical analysis

All experiments were performed at least in triplicate. The data are presented as mean ± SD. Statistical differences between the values obtained in different experimental settings were evaluated by the means of non-parametric Mann-Whitney test (P<0.05) using STATISTICA 6.0 (StatSoft Inc, Tulsa, OK, USA). Curve fitting was performed in OriginPro 8 (OriginLab Corporation, Northampton, MA, USA).

## Results

### Changes of mitochondrial membrane potential in response to fluctuating H_2_O_2_ levels

**Figure 1:**
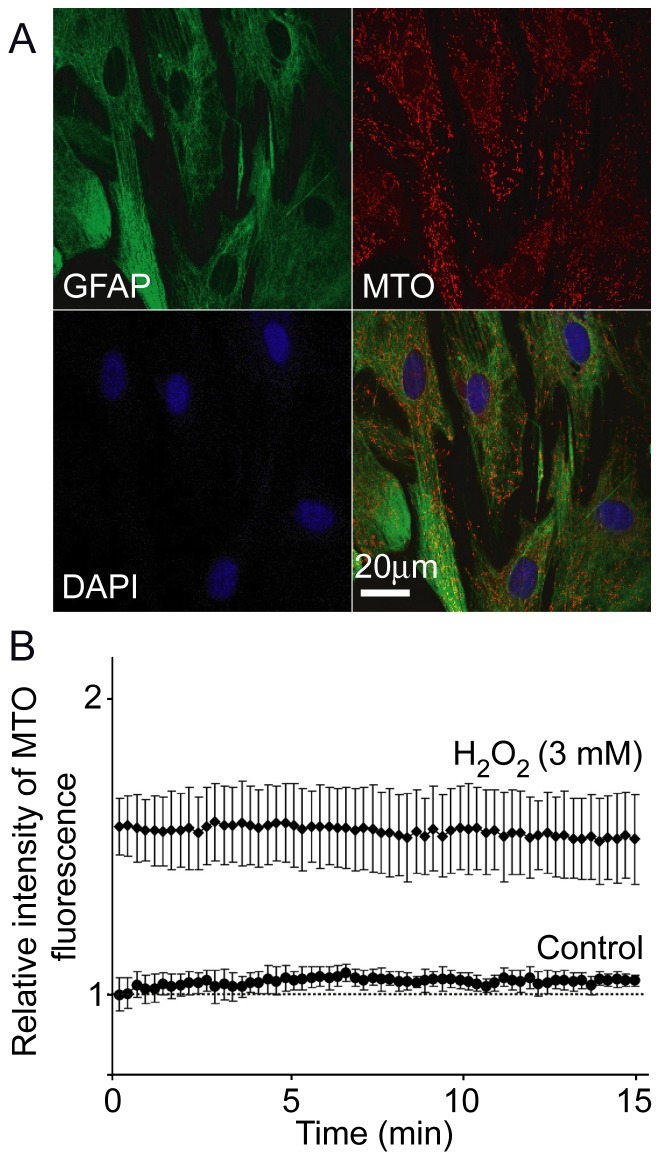
Technical features of primary astrocytes culture and MTO fluorescence. (**A**) Triple staining of astrocytes in culture. Astrocytes were pre-stained with MTO, then fixed and stained with antibodies against GFAP and with nuclear dye DAPI. (**B**) The intensities of MTO fluorescence over time. Graph represents relative average intensities of MTO signal of live control cells and cells exposed to continuous 3 mM H_2_O_2_ (n = 25). Time frame of treatment with H_2_O_2_, MTO staining, post-wash recovery and acquisition were the same as in experiments with continuous and fluctuating exposure to H_2_O_2_.

**Figure 2:**
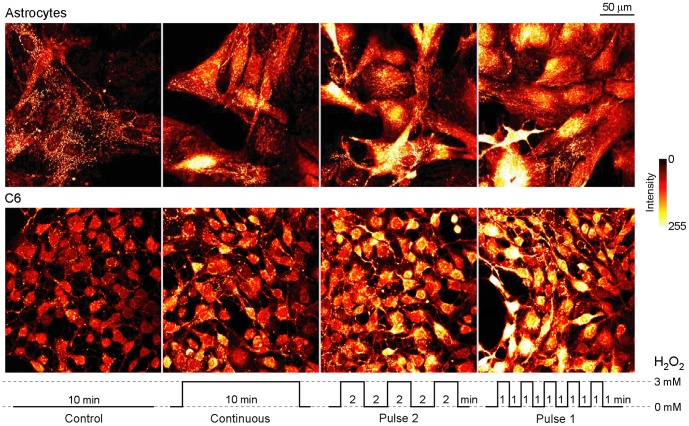
Confocal micrographs of astrocytes and C6 astroglial cells exposed to H_2_O_2_ using three different regimes, and subsequently labeled with MTO. The regimes are illustrated at the bottom. Optical thickness was 3.5 µm.

**Figure 3:**
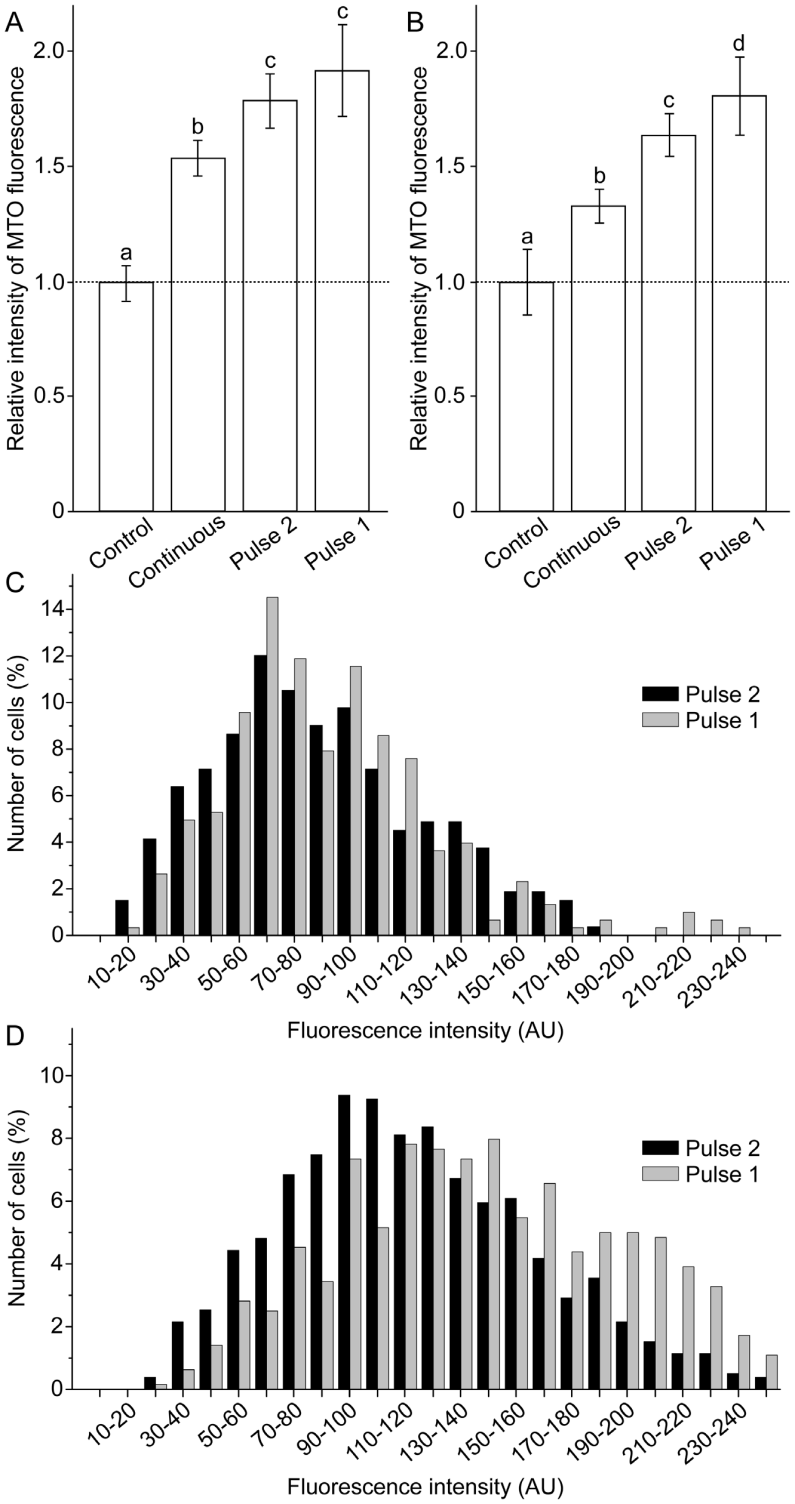
Relative intensity and percentage distribution of MTO fluorescence. (**A**) Relative intensity of MTO fluorescence in astrocytes. (**B**) Relative intensity of MTO fluorescence in C6 cells. Bars not sharing a common letter are significantly different (P<0.05). (**C**) Percentage distribution of astrocytes according to average per-pixel intensities of MTO fluorescence. (**D**) Percentage distribution of C6 cells according to average per-pixel intensities of MTO fluorescence. The number of cells (%) was bracketed to 10 AU steps, covering the whole range of fluorescence intensities (0–255 AU). Treatment regimes are illustrated in Figure 2.

Astroglial culture was characterized by >95% homogeneity of GFAP positive cells, as defined by immunofluorescence labeling (Figure 1A). Control experiments were performed in order to evaluate the stability of MTO signal. Cells were perfused with ECS with or without H_2_O_2_ for 10 min, and dyed with MTO. Representative fields were imaged for 15 min, during which MTO signals were stable (Figure 1B). Figure 2 shows characteristic confocal micrographs of astrocytes and C6 astroglial cells stained with MTO. This dye is a lipophilic cationic fluorescent mitochondrial marker, which enters mitochondria in MMP-dependent manner in order to covalently bind free thiols. Higher MMP denotes a more negative (hyperpolarized) matrix and a higher driving force for MTO diffusion across the inner mitochondrial membrane, ultimately resulting in higher fluorescence intensity [31]. It can be observed that the treatment with H_2_O_2_ caused hyperpolarization in both cell types. Figure 3 represents the quantification of these results. In primary astrocytes, fluctuating H_2_O_2_ induced a more pronounced increase of MMP compared to continuous treatment (Figure 3A). High rate fluctuating regime of exposure – Pulse 1 resulted in higher fluorescence intensity compared to low rate fluctuating sequence of exposure – Pulse 2, but the difference was not statistically significant. A similar trend was obtained for C6 cells (Figure 3B), but the difference between Pulse 2 and Pulse 1 was statistically significant. Differences between MMP in cells exposed to Pulse 2 and Pulse 1 were analyzed further by establishing percentage distribution of cells according to their average per-pixel fluorescence intensities. The acquired data confirmed the results presented in Figures 3A and 3B. Similar distributions can be observed for astrocytes exposed to Pulse 2 and Pulse 1 regimes (Figure 3C), and no statistically significant difference was observed. On the other hand, in comparison to C6 cells treated with Pulse 2 regime, percentage distribution of cells exposed to Pulse 1 was shifted towards higher florescence intensities (Figure 3D), which showed significantly higher values (P = 0.012). Altogether, the rank order of MMP hyperpolarization was Pulse 1 ≥ Pulse 2> continuous exposure > control.

### Mitochondrial activity and ATP level

**Figure 4:**
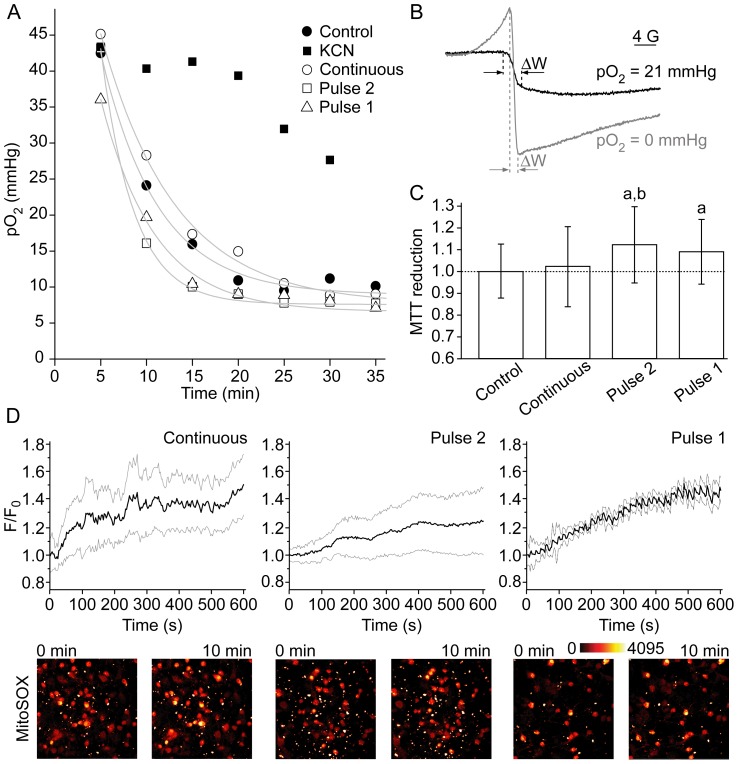
The parameters of mitochondrial activity in C6 cells exposed to H_2_O_2_ using three different regimes. The regimes are illustrated in Figure 2. (**A**) Oxygen consumption during the period of 35 min following the treatment and trypsinization, as determined by the means of EPR oximetry. Cyanide (1 mM) was used to inhibit mitochondrial respiration, in order to test the method. Control: *τ* = 6.22 min; *r* ^2^ = 0.988; Continuous: *τ* = 8.09 min; *r* ^2^ = 0.996; Pulse 2: *τ* = 3.57 min; *r* ^2^ = 0.990; Pulse 1: *τ* = 5.84 min; *r* ^2^ = 0.991. (**B**) EPR spectra of India ink showing different peak-to-peak line widths (ΔW) in water with pO_2_ of 0 and 21 mmHg. (**C**) The relative reduction of MTT (letters over bars indicate significant difference: a – compared to control; b – compared to continuous exposure; P<0.05). (**D**) The relative production of superoxide in mitochondria during the treatment (standard deviation margins are depicted with gray lines). Fluorescence intensity (F) was normalized to baseline per-pixel intensity (F_0_) for each cell (n = 20). Presented micrographs were obtained at the start (0 min) and at the end of the treatment (10 min).

**Figure 5:**
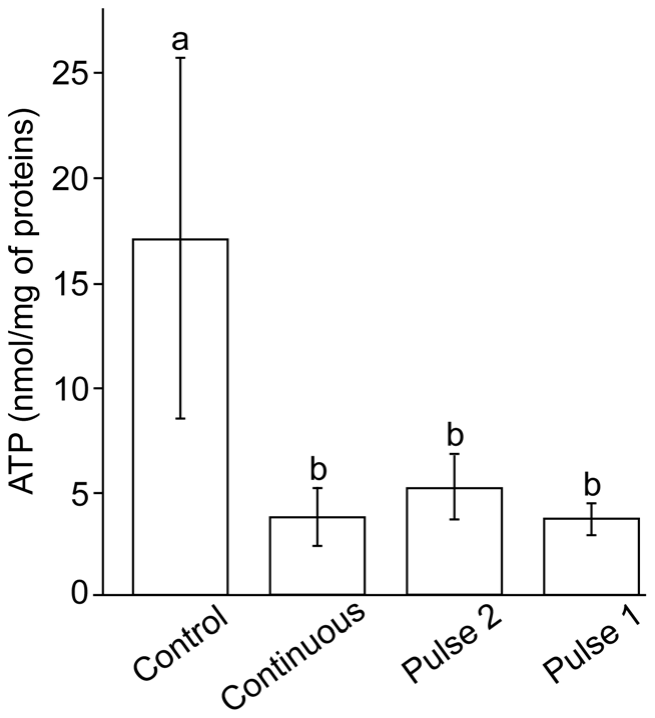
Intracellular concentration of ATP in C6 cells exposed to H_2_O_2_ using three different regimes. The regimes are illustrated in Figure 2. Bars not sharing a common letter are significantly different (P<0.01).

The established phenomenon was investigated further only on C6 cells, because of the limits posed by the low yield of primary astrocytes. Figure 4 shows that the activity of ETC was not significantly affected by H_2_O_2_. No statistically significant differences were observed for the consumption of O_2_ by C6 cells exposed to different treatment sequences (Figure 4A), although cells exposed to Pulse 2 appear to exhibit a slightly promoted respiration. Oxygen tensions were calculated from the line widths of India ink spectra (Figure 4B). MTT reduction was promoted in cells exposed to Pulse 2 and Pulse 1 treatment sequences (Figure 4C). The reduction of MTT is provoked by ETC electron carriers [32], but also by mitochondrial, cytoplasmic-soluble and membrane-bound oxidoreductases, and small molecular weight reductants [33]. In our experimental settings, the effects of small cytoplasmic reductants could be written off, because H_2_O_2_ can be expected to decrease their level, as documented here by the drop in thiol level. However, oxidoreductases might be at least partially responsible, particularly if we take into account that some preliminary results imply that astrocytes increase NADH level in response to H_2_O_2_ [34]. Different regimes of exposure to H_2_O_2_ provoked a similar level of superoxide production in mitochondria during the treatment, as determined by the means of time-lapse confocal microscopy and superoxide-sensitive mitochondria-specific fluorescent probe MitoSOX (Figure 4D). Figure 5 shows the changes of intracellular ATP concentrations in C6 cells. The treatment with H_2_O_2_ resulted in drastic decrease of ATP level, regardless of the regime of exposure.

### Intracellular calcium level fluctuates with H_2_O_2_

**Figure 6:**
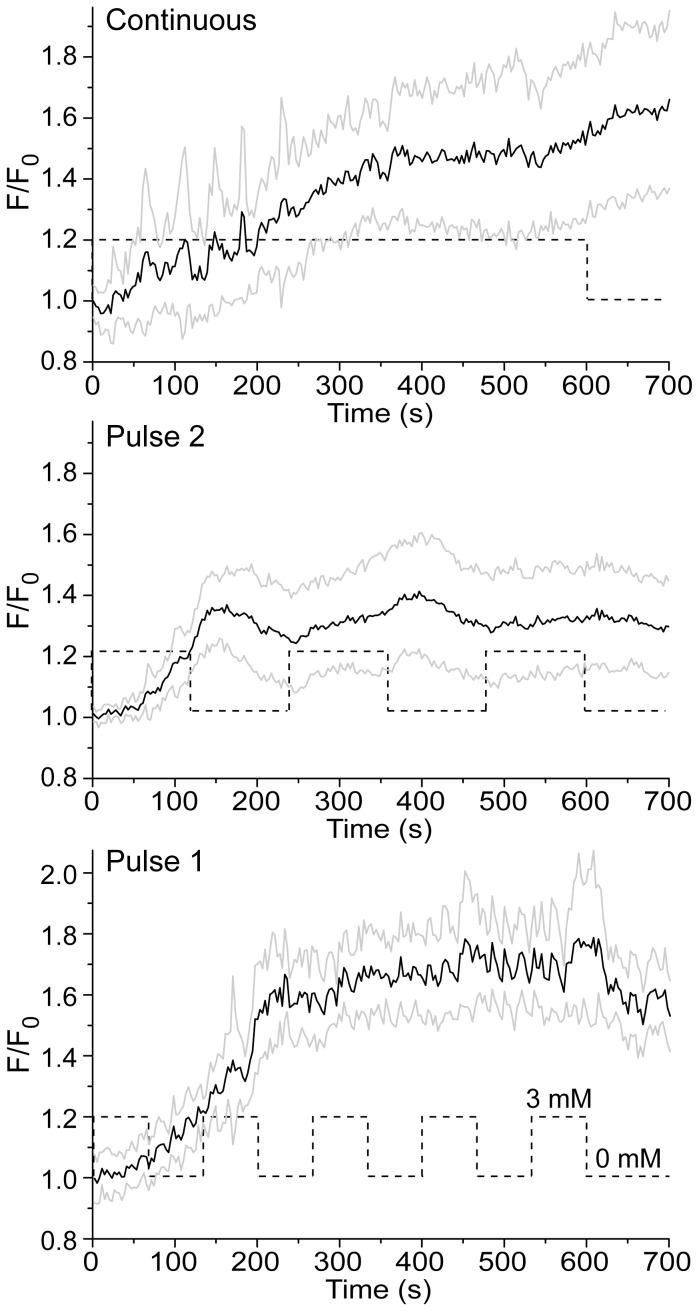
The changes of [Ca^2+^]_i_ in C6 cells exposed to H_2_O_2_, as determined using calcium-sensitive probe Fluo-3. Standard deviation margins are depicted with gray lines. Fluorescence intensity (F) was normalized to baseline per-pixel intensity (F_0_) for each cell (n = 20). Dashed lines illustrate treatment regimes.

Figure 6 shows that H_2_O_2_ causes the increase of [Ca^2+^]_i_, which is in accordance with previous reports [20]. Fluctuating oxidative stress provokes a specific pattern of large-amplitude [Ca^2+^]_i_ fluctuations, which follows the pattern of exposure to H_2_O_2_. A remarkable correlation of trends in [Ca^2+^]_i_ and the treatment sequence can be observed for Pulse 2 experiments. A correlation is less obvious for Pulse 1, most likely due to a lag in the response of [Ca^2+^]_i_ to H_2_O_2_ (which is also present in Pulse 2), combined with a higher rate of H_2_O_2_ fluctuations. It is noteworthy that the resulting [Ca^2+^]_i_ was slightly lower for Pulse 2 compared to continuous regime and Pulse 1, but no statistical significance was determined at the end of the treatment (10 min).

### Nuclear translocation of NF-κB and thiol level

**Figure 7:**
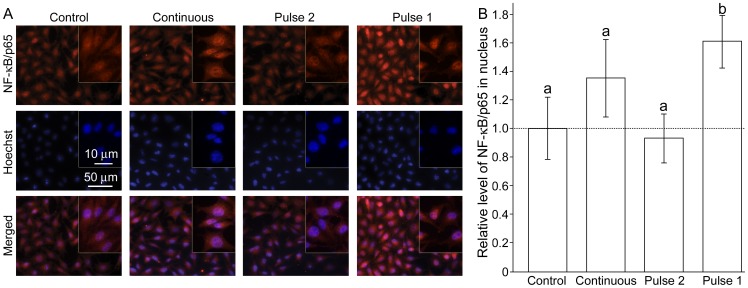
The nuclear level of transcription factor NF-κB in C6 cells exposed to H_2_O_2_ using three different regimes. The regimes are illustrated in Figure 2. (**A**) Micrographs showing NF-κB immunofluorescence (top), nuclei stained with Hoechst (middle), and the merged images (bottom). (**B**) The relative level of nuclear NF-κB/p65. Bars not sharing a common letter are significantly different (P<0.05).

The nuclear level of NF-κB/p65 was determined 2 hours after the treatment, because it is known that NF-κB responds to H_2_O_2_ rather slowly (in hours range) [2]. The significant activation was observed only for Pulse 1 (Figure 7). In comparison to constitutive NF-κB activity in controls, the continuous exposure to H_2_O_2_ appears to provoke activation, but it was slightly out of the limits of statistical significance (P = 0.078). Pulse 2 did not provoke any activation at all.

**Figure 8:**
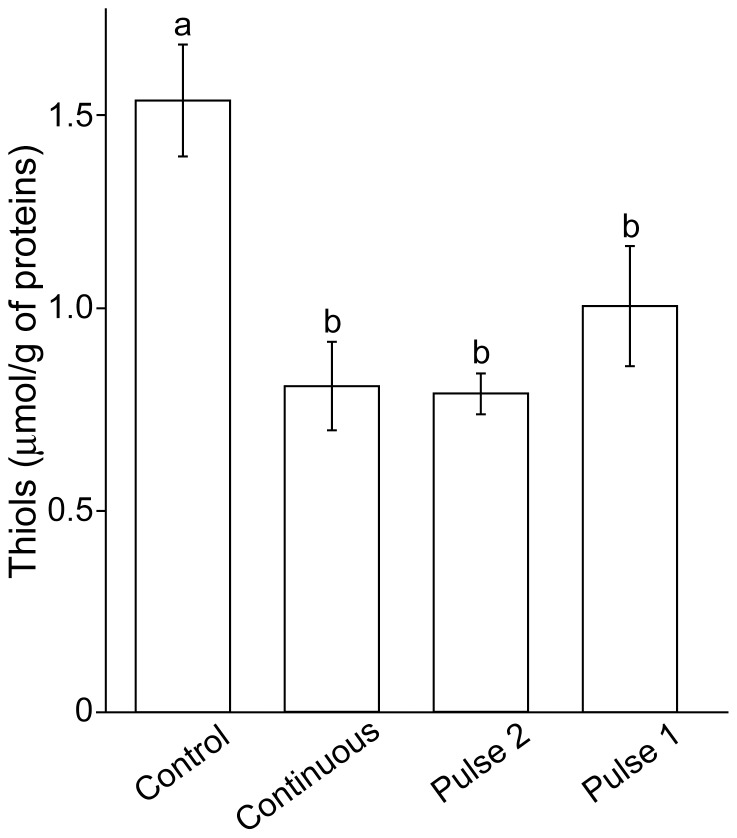
The level of thiol groups in C6 cells exposed to H_2_O_2_ using three different regimes. The regimes are illustrated in Figure 2. Bars not sharing a common letter are significantly different (P<0.05).

It was important to determine the resulting levels of oxidation, since the total time of exposure to H_2_O_2_ for continuous regime, Pulse 2, and Pulse 1 are different (10, 6, and 5 min, respectively). It can be observed that free thiol levels have decreased in all samples exposed to H_2_O_2_, independently of the regime of exposure (Figure 8). This implies that the observed differences in the effects of continuous *vs.* fluctuating H_2_O_2_ are not related to the blunt decrease of the number of free thiol groups.

## Discussion

Our study introduces fluctuating oxidative stress as a novel redox parameter that takes into account dynamic (redox) regime in which cells operate, particularly under pathophysiological conditions [35]. We examined the effects of three regimes of exposure to increased H_2_O_2_ concentration – continuous, fluctuating at low rate, and fluctuating at high rate, on the set of redox-sensitive parameters in C6 astroglial cells and primary astrocytes. MMP, [Ca^2+^]_i_, NF-κB, and reducing capacity responded differently to fluctuating oxidative stress than to the continuous exposure, whereas other parameters, *i.e.* [ATP]_i_, respiration rate, and superoxide production in mitochondria, did not. The observed effects of continuous exposure to H_2_O_2_ are in accordance with previously published results on astroglial cells showing ATP depletion [18], [19], Ca^2+^ mobilization and influx [20], and MMP hyperpolarization [21]–[23].

Although not directly examining the phenomenon of fluctuating oxidative stress, some previous studies imply that the effects of fluctuations of H_2_O_2_ level or some other redox-relevant parameters might differ compared to a continuous regime. For example, one behavioral study on *Drosophila* has shown that H_2_O_2_ causes activity increase and changes in behavior [36]. However, flies fed with H_2_O_2_-containing medium (resulting in fluctuating H_2_O_2_ body level) responded differently compared to transgenic flies with increased intrinsic H_2_O_2_ production (analogous to the continuous exposure). The former showed more erratic behavior and suppressed daily locomotor rhythms, while the latter exhibited faster movements but intact daily locomotor rhythms. Furthermore, it has been reported that in comparison to steady laminar shear stress, oscillatory shear stress causes the development of drastically pronounced pro-oxidative conditions in endothelial cells [37]–[39]. Pertinent to our study, pulsatile shear stress induced a time-dependent MMP increase in human umbilical cord endothelial cells. The effect was attenuated when MnSOD activity was suppressed, implying the role of H_2_O_2_ in the development of hyperpolarization [39].

The nuclear level of H_2_O_2_-regulated transcription factor NF-κB is known to oscillate within one cell upon activation [2], [35], [40]. The final effects of NF-κB signaling might depend on frequency, number, and amplitude of oscillations [5], [41]–[43]. On the other hand, continuous stimulation and different patterns of pulsatile stimulation of NF-κB system lead to different patterns of NF-κB-dependent gene expression [41]–[43]. We found that NF-κB activation depends on the regime of exposure to H_2_O_2_. Importantly, in comparison to many other cell types, astrocytes show high constitutive expression of NF-κB, which appears to play a vital role in the resistance of these cells to oxidative stress [44]. One study has shown that a prolonged exposure of astrocytes to H_2_O_2_ (h/mM range) causes a gradual decrease of DNA binding and transcriptional activity of NF-κB [44]. However, it appears that the response of NF-κB to H_2_O_2_ in astroglial cells is much more complex, and depends on time, concentration, and the regime of exposure. It is possible that H_2_O_2_ fluctuations impact various metabolic pathways via Ca^2+^ and NF-κB signaling.

Presented results reflect on some other important issues. To be specific, the effects of oxidative stress might be overestimated in studies that are conducted using cultured cells due to: (i) altered (redox) metabolism of cultured cells compared to cells in living tissues; and (ii) the presence of redox-active metals and some other pro-oxidative components (even H_2_O_2_) in cell culture media [45]. In contrast, it appears that *in vitro* studies may sometimes underestimate the *in vivo* effects of oxidative stress, as these do not take into account the variability of H_2_O_2_ concentrations, which might be present under pathophysiological settings in cells and tissues.

In conclusion, the effects of H_2_O_2_ on cellular/mitochondrial metabolism do not only depend on the concentration and time of exposure, but also on the fluctuations of H_2_O_2_ level. This raises a large number of interesting questions, such as: How cells discriminate between continuous and fluctuating H_2_O_2_ levels? and What might be the potential role of these fluctuations in cellular (patho)physiology? These require further investigations, but some speculations could be made based on available data. Hydrogen peroxide represents a pleiotropic signaling species, which modulates the activity of a large number of proteins – enzymes, transcription factors, channels, and others [3]. Thiol (-SH) residues in proteins are key H_2_O_2_-sensitive redox switches in mammalian cells. Thiol groups act as four-step switches, being gradually oxidized by H_2_O_2_ to sulfenic (-SOH), sulfinic (-SO_2_H) and sulfonic (-SO_3_H) acids. The first step of oxidation is reversible, while sulfinic and sulfonic modifications are considered to be irreversible (although there are data showing that the former might be reversible after all [46]). It can be assumed that continuous exposure to H_2_O_2_ (*i.e.* prolonged pro-oxidative conditions) potentiates the formation of irreversible modifications. In contrast, shorter pulse exposures result predominantly in the formation of -SOH groups, which can be reversed back to -SH by glutathione and other reducing agents. In addition, different fluctuation rates might result in specific profiles of thiol modifications that encode divergent signals and responses. Therefore, cells might discriminate between continuous and fluctuating H_2_O_2_ levels (as well as between different fluctuation rates) via: (i) Different levels of specific modifications; (ii) Distinct effects of -SOH, -SO_2_H, and -SO_3_H modifications on the activity of target proteins; (iii) Different effects of reversible and irreversible modifications. It is known that H_2_O_2_ activates adaptive mechanisms which increase the resistance to subsequent exposure to the same stimulus (hormesis) [3]. Hence, if cells are facing consistent pro-oxidative conditions, they are stimulated to respond and adapt. However, if the conditions are varying, cells are stimulated only to respond, and not to activate costly adaptive mechanisms, because it is still not certain whether the adaptation is needed. For example, T cells have thiol redox switches on their surface. T cells respond to -SOH modifications by decreasing the activity, which can be re-established in the presence of glutathione-releasing dendritic cells. However, excessive thiol oxidation to -SO_3_H has a more permanent and irreversible effect - apoptosis [17]. In addition to their role in the response to extracellular stimuli, fluctuations might be involved in strictly intracellular processes. For example, mitochondria represent the crossroad between energy and redox metabolism, since ETC is the main generator of both ATP and superoxide – the precursor of H_2_O_2_. The production of superoxide and H_2_O_2_ is defined by specific modes of ETC activity [7], so the emitting rate of H_2_O_2_ most likely carries the information about mitochondrial metabolism. Pouvreau has shown recently that clusters of mitochondria act as synchronized units that spontaneously generate superoxide flashes [8]. Consequent fluctuations of H_2_O_2_ level might be involved in orchestrating the clusters. In addition, fluctuations might invoke necessary adjustments in the microenvironment in order to meet the requirements for optimal mitochondrial activity. Finally, the rate of H_2_O_2_ release from the cell most likely reflects the dynamics of cellular metabolism, providing neighboring cells with information about local (patho)physiological settings.
